# Changes in periodontal parameters of splinted versus non-splinted posterior teeth during ten years of supportive periodontal therapy – A retrospective evaluation

**DOI:** 10.1007/s00784-024-05679-2

**Published:** 2024-04-29

**Authors:** Sarah K. Sonnenschein, Samuel Kilian, Maurice Ruetters, Antonio Ciardo, Ti-Sun Kim

**Affiliations:** 1 Heidelberg Faculty of Medicine, Department of Conservative Dentistry, Heidelberg University, Clinic for Oral-, Dental- and Maxillofacial Diseases, Im Neuenheimer Feld 400, 69120 Heidelberg, Germany; 2 Heidelberg University, Institute of Medical Biometry, Heidelberg, Germany

**Keywords:** Splinting therapy, Tooth mobility, Periodontal therapy, Fibre-reinforced-composite splint, Supportive periodontal therapy

## Abstract

**Objectives:**

To compare periodontal parameters of splinted posterior teeth versus control teeth over ten years of supportive periodontal therapy (SPT) and to assess the survival rate of splints.

**Material and methods:**

Retrospective data of 372 SPT-patients was screened for splints (composite/fiberglass-reinforced composite) in the posterior (molars/premolars) which were inserted at least ten years before due to increased tooth mobility. For each splinted tooth (test), a corresponding control tooth had to be present at the first SPT-session after splint insertion (T1). Data was assessed at T1 and ten years later (T2). Possible influencing covariates for splint survival (mobility degree/Eichner class) were tested by Cox regression. The change in clinical attachment level (ΔCAL), probing pocket depth (ΔPPD) and the testing of possible influencing covariates was analyzed by using mixed linear regression.

**Results:**

Twenty-four patients (32 splints, 58 splinted teeth) were included. Ten test and two control teeth were lost. No differences were observed between ΔCAL and ΔPPD of test teeth compared to control teeth (ΔCAL -0.38 ± 1.90 vs. 0.20 ± 1.27 mm; ΔPPD -0.17 ± 1.18 vs. 0.10 ± 1.05 mm). Twenty-two splints fractured during the observation period (survival-rate: 31%). Mobility degree and Eichner class did not influence time until fracture.

**Conclusions:**

Splinting of periodontally compromised and mobile posterior teeth does not have any disadvantage regarding the clinical periodontal situation when regular SPT is applied. However, splint fractures occur very often.

**Clinical relevance:**

Splinting of posterior teeth is a treatment option in addition to active periodontal therapy when patients are disturbed by tooth mobility but splints have a high susceptibility to fracture.

## Introduction

A common symptom of advanced periodontitis is increased tooth mobility [1]. Usually, increased tooth mobility related to periodontitis is the result of various pathological changes, which are often present in combination in patients with severe periodontitis. These pathological changes include acute periodontal inflammation and associated laxity of periodontal supportive tissues, the apical shift of the center of rotation of the tooth due to advanced attachment and alveolar bone loss as well as traumatic occlusion.

In many cases, pathological tooth mobility due to periodontitis can be reduced or even eliminated by systematic periodontal therapy, including the elimination of periodontal inflammation and the correction of occlusal pre-contacts. However, in cases of severe attachment loss but stable periodontal conditions after treatment, persisting tooth mobility may be a problem resulting from the irreversible apical shift of the center of rotation of the tooth.

This may affect the patient’s chewing ability, phonetic skills, oral comfort [2, 3] and the Oral Health-Related Quality of Life (OHRQoL) in general [4]. A simple procedure to reduce the pathological mobility of periodontally damaged teeth is the adhesive connection of affected teeth to the adjacent teeth by composite or glass-fiber-reinforced splinting.

Unfortunately, there is still limited evidence on the long-term effects of splinting therapy of periodontally damaged and mobile teeth on periodontal stability and survival rates of teeth treated this way.

Although the few studies on this subject indicate high survival rates of splinted teeth and long-term periodontal stability during SPT [4–7] most of them focus on mandibular incisors [6–8].

For the survival rate of splints (until fracture/need for repair), different results are shown, ranging from very frequent fractures [3, 5] up to high survival rates of splints in the mandibular front of 95% after 4.5 years [6], 65.2% after 4.55 years [7], and 67% after 10 years [8]. In this context, a recent study indicates that the fracture probability of a splint is influenced by its position in the jaw [5]. Based on these indications and because the loads and shearing forces to which teeth or splints are exposed to within the oral cavity are known to vary greatly depending on their position in the jaw, a more differentiated approach to evaluate periodontal stability and survival rates of splinted teeth as well as splint survival makes considerable sense.

Therefore, the aim of this study is to compare the periodontal parameters and survival rate of splinted posterior teeth with non-splinted control teeth over a ten-year period of supportive periodontal therapy (SPT) and to assess the survival rate of posterior splints.

## Materials and methods

### Screening of patients

Retrospective data (dental records and radiographs) of 372 adult patients who had a SPT-session between July 2014 and January 2016 in the authors department and had agreed to participate in a study on different aspects of SPT and long-term survival of periodontally compromised teeth (German Clinical Trials Register DRKS00011316) was screened for the presence of composite and fiberglass-reinforced composite splints in the posterior region (molars and premolars). After identifying all patients in the study cohort with splints in the posterior region, they were screened for fulfillment of the patient-specific inclusion criteria. Within these patients, only those splints and splinted teeth were included which met the criteria at splint- and tooth-level.

### Inclusion and exclusion criteria at patient- and splint-level

The following inclusion criteria are established at patient-level:

- Patient has been treated for periodontitis at the authors’ institution according to the established regimen of systematic periodontal therapy (as described in Sonnenschein et al. 2020 [9]).
- After completion of the active periodontal treatment, the patient attended SPT at least once per year for ten years or longer.
- Complete dental and periodontal findings at the defined observation time points.

The following inclusion criteria are established at splint-level:

- Presence of a composite or fiberglass-reinforced composite splint in the posterior region (premolars or molars).
- The splint was placed due to increased mobility of at least one periodontally damaged tooth.
- If fracture or debonding of the splint occurred within the defined observation period, the test tooth was reintegrated into the splint-connection by repairing the splint.

### Defining the test and control tooth

Each mobile tooth integrated into the splint is defined as a "test tooth". The tooth mobility was determined according to Lindhe & Nyman (degree I: mobility in in labio-oral direction of 0.2–1 mm, degree II: mobility of 1–2 mm, degree III: exceeding 2 mm in labial-oral direction and or in vertical direction). A "control tooth" is defined for each test tooth. The contralateral same tooth of the same patient serves as the preferred control tooth. If the direct contralateral tooth is not available or must be excluded, (1) a contralateral tooth of the same tooth type or (2) a tooth of the same type from the opposite jaw serves as control.

The following inclusion criteria are set at test tooth-level:

- Molar (first or second) or premolar integrated in a composite or fiberglass-reinforced composite splint with a clinical attachment level (CAL) ≥ 4 mm at least at one site.
- At T1, a control tooth is available for the respective test tooth.
- The test tooth was not treated regeneratively or resectively before and during systematic periodontal therapy or between T1 to T2.

The following inclusion criteria are set at the control tooth-level:

- The control tooth was not treated regeneratively or resectively before and during systematic periodontal therapy or between T1 to T2.
- Third molars are excluded as controls.

### Observation time points and obtained data

The first SPT-session after splint insertion is defined as the baseline findings (T1). The SPT-session ten years after T1 (± 18 months) is defined as the ten-year findings (T2).

Complete documentation of dental and periodontal status included the detailed findings of all existing teeth and the recording of periodontal pocket depth (PPD) and CAL at six sites per tooth (mesio-buccal, centro-buccal, disto-buccal, mesio-oral, centro-oral, disto-oral) with assessment of bleeding on probing at each site (BOP: yes/no), mobility degree (according to Lindhe & Nyman [10]) and furcation involvement (according to Hamp et al. [11]).

At patient-level, the following data are assessed: age (in years), sex (male/ female), initial periodontal diagnosis (stage and grade according to the AAP/ EFP classification of periodontal diseases [1]), Eichner class [12] at T1 and T2, presence of systemic factors (present/ absent), smoking status (smoker/ non-smoker), adjunctive antibiotic administration as part of active periodontal therapy (prescribed/ not prescribed), individual periodontal risk assessment (low/ moderate/ high risk) [13], and total number of teeth and implants.

At splint-level, the following data are assessed: number of splinted teeth, number of splint-blocks per patient, type of splint (composite splint/ fiberglass-reinforced composite splint; type of composite and fiberglass), fracture/ debonding of splint (months to event), number of splint fractures during the observation period. Information on splints is obtained by reviewing the patient’s charts.

At tooth-level, the following parameters are collected: PPD, CAL, BOP, gingival bleeding index (GBI) [14], plaque control record (PCR) [15] (at T1 and T2, for test/ control teeth and the complete dentition), mobility degree [10] prior to splinting (for test and control teeth), maximum degree of furcation involvement [11] (for test and control teeth), presence of an antagonist (for test and control teeth), and tooth loss during the observation period. In case of tooth loss, the reason was documented, if available. The tooth-level information is obtained from the detailed dental and periodontal findings at T1 and T2 and by reviewing the patient’s charts. All participants were examined within a regular SPT session by calibrated dentists (as described in Sonnenschein et al. 2020 [9]).

### Relative alveolar bone loss

If available, the relative alveolar bone loss (rABL) at the test and control teeth is assessed from radiographs (orthopantomogram or periapical radiograph of the corresponding teeth) at T1 and T2 (± 18 months) by an experienced and trained examiner (M.R.). The rABL is assessed at the most affected sites of the test- and control-teeth and calculated by dividing the distance (in mm) from the bottom of the bony defect to the cemento-enamel junction or the (single tooth) crown margin by the distance between the cemento-enamel junction or the crown margin to the radiographic apex of the root. The measurement of the radiographs was performed digitally (software: Sidexis XG 2.63, Dentsply Sirona Inc., York, USA).

### Statistical analysis

As this study is explorative, no formal sample size calculation was performed. The recorded periodontal status are entered manually into the same tabular program independently by two different persons (J.L.; S.K.S.) from the dental and periodontal records of the patients. Any discrepancies were corrected accordingly after the original documents were reviewed again. Periodontal parameters (CAL, PPD, changes in CAL and PPD, BOP) and oral hygiene parameters (GBI, PCR) are described by mean ± standard deviation, median, first and third quantile, minimum, and maximum.

Mixed linear regression was applied at tooth-level to analyze influencing factors on the outcome variables CAL difference and PPD difference between T2 and T1. Included covariates were CAL (or PPD) at T1, mobility degree, maximum furcation involvement, presence of systemic diseases, smoking status, and presence of antagonistic contacts. Patient ID was included as random intercept to adjust for the dependence structure of multiple teeth within the same patient. Missing values occurred in the variables CAL and PPD at T2 due to the loss of tooth. They were handled by multiple imputation using the R package mice [16] with 100 multiple imputed data sets and 20 iterations. The imputation method was “Predictive Mean Matching”, using the variables age, smoking status, systemic diseases, CAL and PPD at T1 and T2, maximum mobility degree, maximum furcation involvement, antagonistic contact, and group (test/ control). As a sensitivity analysis, mixed linear regression was also performed on the complete cases without multiple imputation.

The survival rate of splints until fracture, debonding or need for repair is visualized by Kaplan–Meier curves with 95% CI. The splint survival is reported from splint insertion (which may have been prior to T1) until T2. Cox regression was used to determine the association between the survival of splints and the possible predictors supporting zones (Eichner class) and mobility degree. Third molars and dental implants were excluded from analysis. All p-values are to be interpreted descriptively, thus no adjustment for multiple testing was done. P-values below 0.05 were regarded as considerable. Analysis was done using the statistical software R v. 4.0.1 (The R Project, The R Foundation).

## Results

### General description of the study cohort

**Fig. 1:**
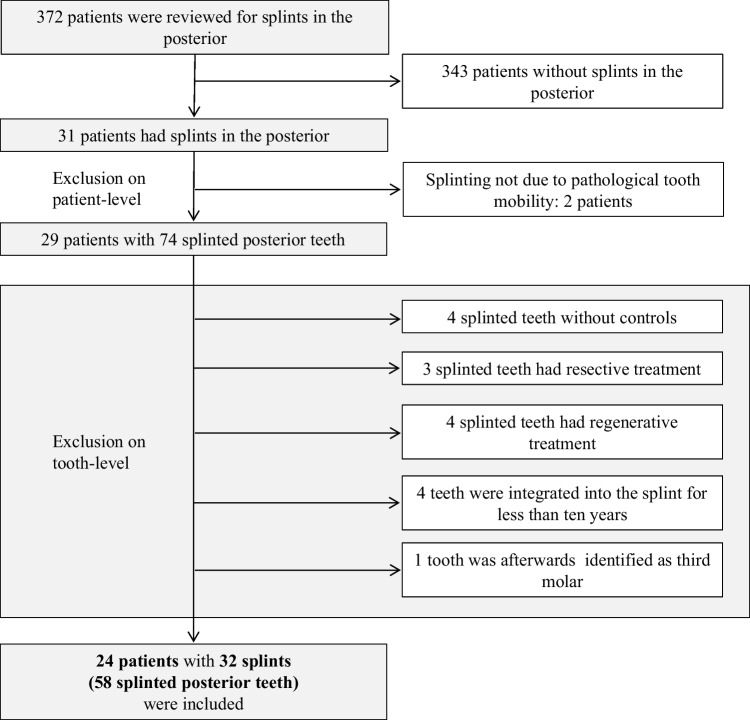
Flowchart for application of inclusion and exclusion criteria at patient-, splint-, and tooth-level

A total of 24 patients (14 female; 10 male) met all inclusion criteria at the patient-, splint-, and tooth-level. The included patients had a total of 58 test-control tooth pairs that could be included. At the splint-level, 32 splints (in which the 58 test teeth were integrated) were included. Figure 1 shows the flowchart for applying the inclusion and exclusion criteria at all levels. The mean age of the patients at T1 was 50 ± 7.8 years (range: 38–68; median: 50). The initial periodontal diagnosis was stage III periodontitis in seven patients (29.2% of all patients, including three patients with grade B and four patients with grade C) and stage IV periodontitis in 17 patients (70.8% of all patients, including two with grade B and 15 with grade C).

At T1, ten patients were smokers but three of them had quit smoking at T2 (≥ 5 years non-smokers at T2). Two patients suffered from diabetes mellitus. According to the Eichner classification, 16 patients (66.7%) were class A (all four supporting zones present), seven patients (29.2%) were class B (two supporting zones present in five patients; one supporting zone present in two patients), and one patient (4.2%) was class C (one jaw edentulous/no supporting zone present). At T1, four patients had a low individual periodontitis risk profile (16.7%), 13 a moderate-risk profile (54.2%) and seven a high-risk profile (29.2%). The number of patients with implants increased from three at T1 (6 implants in total; range: 1–4) to seven at T2 (21 implants in total; range: 1–6). The dental and periodontal situation of the overall dentition of the study cohort is shown in Table 1.

**Table 1:** Descriptive statistics [mean ± standard deviation (range; median)] for the dental and periodontal situation of the overall dentition (patient-level) of the study cohort at baseline (T1) and after ten years of supportive periodontal therapy (T2)

	T1	T2
teeth [number]	22.5 ± 4.8 (9–28; 24)	21.5 ± 4.9 (9–27; 23)
PPD [mm]	2.54 ± 0.41 (1.50–3.24; 2.58)	2.48 ± 0.41 (1.92–3.57; 2.39)
CAL [mm]	4.36 ± 1.09 (2.25–6.55; 3.98)	4.43 ± 0.98 (2.27–6.11; 4.49)
BOP [%]	11.0 ± 6.8 (0–28; 10.08)	13.5 ± 8.6 (0–36.6; 12.6)
GBI [%]	2.8 ± 4.8 (0–24; 2.0)	1.9 ± 3.1 (0–10; 0.0)
PCR [%]	29.4 ± 13.1 (5–59; 26.5)	31.7 ± 10.9 (8–50; 33.0)

Teeth, number of teeth in the overall dentition; *PPD* periodontal pocketing depth for the overall dentition [mm]; *CAL* clinical attachment level for the overall dentition [mm]; BOP, bleeding on probing for the overall dentition [%]; *GBI* gingival bleeding index [14] for the overall dentition [%]; *PCR* plaque control record [15] for the overall dentition [in %]

#### Tooth loss and change of periodontal parameters at tooth- level

Ten test teeth and two control teeth were lost during the ten-year SPT observation period, resulting in a statistically significant difference (p = 0.029, Fischer’s exact test). The reasons for tooth loss in the test teeth were due to endodontic complication (three teeth), root fracture (two teeth), perio-endodontal lesion with abscess formation (two teeth), caries (one tooth), periodontal reason (one tooth), and root amputation (one tooth; need for resective treatment during the observation period led to exclusion but the tooth was still in situ at T2). The two control teeth were lost due to root fracture and periodontal reasons.

**Fig. 2:**
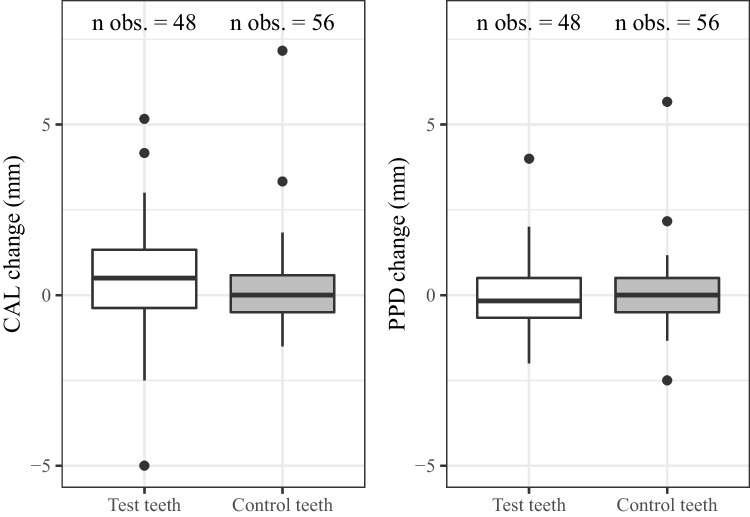
Boxplots of CAL change and PPD change in test and control teeth (ΔCAL; ΔPPD). PPD, periodontal pocket depth [mm]; CAL, clinical attachment level [mm], n obs.; number of observations

Radiographs were available for 31 test-control tooth pairs to determine rABL at T1 and T2. The mean rABL of test teeth was 54% ± 16% (range: 28%—87%; median: 57%) at T1 and 56% ± 15% (range: 24%—100%; median: 54%) at T2. The mean rABL of control teeth was 43% ± 16% (range: 22%—83%; median: 42%) at T1 and 45% ± 17% (range: 22%—86%; median: 44%) at T2. The distribution of the maximum mobility degrees and the maximum furcation involvement is listed in Table 2. The periodontal parameters CAL, PPD and BOP, as well as the plaque scores for the test and control teeth are given in Table 3. The changes in mean CAL and mean PPD (differences from T1 to T2) over the observation period of ten years of SPT are shown in Fig. 2. The multiple regression model after multiple imputation shows an influence of CAL and PPD at T1 on the change in CAL and PPD during ten years of SPT (ΔCAL, ΔPPD) with regression coefficients of -0.476 (CAL) and -0.591 (PPD). Teeth with maximum furcation involvement of degree III had a considerably higher ΔCAL than teeth with a maximum furcation involvement of degree I (mean difference 2.297). All other tested variables show no statistical influence (Table 4). The multiple regression model on complete cases without multiple imputation shows similar results.

**Table 2:** Distribution of the maximum mobility degrees and the maximum furcation involvements [10] for test and control teeth at baseline (T1). N = 58 test and control teeth, each

	Distribution of the maximum mobility degrees and the maximum furcation involvements [10] for test and control teeth at baseline (T1). N = 58 test and control teeth, each	Number of test teeth	Number of control teeth
Mobility degree[10]	No pathological mobility	2	33
	Mobility degree I	12	19
	Mobility degree II	25	6
	Mobility degree III	19	0
Maximum furcation involvement[11]	Teeth without furcation	41	40
	No soundable furcation involvement	6	7
	Furcation: degree I	3	7
	Furcation: degree II	6	2
	Furcation: degree III	2	2
Antagonistic contact	Present	50	48
	Not present	8	10

**Table 3:** Periodontal parameters clinical attachment loss (CAL), periodontal probing depth (PPD), bleeding on probing (BOP), and presence of dental plaque at test and control tooth-level. Number of test teeth at T1: N = 58; number of test teeth at T2: N = 48; number of control teeth at T1: N = 58; number of control teeth at T2: N = 56; BOP was collected at six sites per tooth [yes/no]; plaque was recorded at 4 sites per tooth [yes/no]; ΔCAL, progression of clinical attachment level between T1 and T2, T1, baseline; T2, after ten years of supportive periodontal therapy

		Test teeth	Control teeth
Mean PPD [mm]	T1	3.04 ± 0.89 (1.33 – 5.00; 2.83)	2.57 ± 0.68 (1.33 – 5.00; 2.50)
	T2	2.92 ± 1.01 (1.67 – 8.00; 2.75)	2.63 ± 1.00 (1.67 – 9.00; 2.50)
Mean CAL [mm]	T1	5.37 ± 1.62 (1.83 – 9.33; 5.33)	4.26 ± 1.59 (2.00 – 8.00; 4.17)
	T2	5.52 ± 1.69 (2.33 – 10.83; 5.50)	4.38 ± 1.66 (2.17 – 11.33; 4.25)
BOP [total number of bleeding sites]	T1	56	37
	T2	60	48
Plaque [total number of sites with plaque]	T1	75	72
	T2	76	77
Teeth with at least one site PPD ≥ 5 mm [number]	T1	22	8
	T2	18	8
Teeth with at least one site ΔCAL ≥ 2 mm [number]	T1 – T2	34	27

**Table 4:** Multiple regression analysis of CAL change and PPD change over ten years of supportive periodontal therapy at tooth-level (test/control teeth). Number of test and control teeth at T1 was N = 58, each. Missing data at T2 due to tooth loss (ten test teeth, two control teeth) was handled by multiple imputation. PPD, mean periodontal pocket depth at tooth-level [mm]; CAL, clinical attachment level at tooth-level [mm]; systemic diseases affecting the periodontal situation were defined as diabetes mellitus, rheumatoid arthritis, osteoporosis, and conditions/diseases that lead to immunosuppression. The mobility degree is given according to Lindhe & Nyman[10] (for test teeth mobility prior to splinting, for control teeth mobility at T1). The maximum furcation involvement degree is given at T1 and according to Hamp et al.[11]. Coefficients of categorical variables refer to the comparison to the reference category (see material and method section)

Variable	CAL change T1 to T2			PPD change T1 to T2		
	Estimate	Std. Error	P-value	Estimate	Std. Error	P-value
(Intercept)	1.807	0.589	0.003	1.228	0.427	0.005
Group of test teeth	0.205	0.372	0.583	-0.215	0.235	0.363
Mean CAL at T1/ Mean PPD at T1	-0.476	0.095	< 0.005	-0.591	0.131	< 0.005
Mobility degree I	0.151	0.367	0.682	0.219	0.235	0.354
Mobility degree II	0.361	0.445	0.419	0.508	0.288	0.080
Mobility degree III	0.752	0.558	0.181	0.383	0.359	0.289
Presence of systemic diseases (T1)	0.383	0.659	0.563	0.001	0.474	0.998
Smoking at T1	0.084	0.264	0.752	-0.263	0.190	0.168
Presence of an antagonistic tooth	0.299	0.411	0.468	0.408	0.261	0.122
Maximum degree of furcation: I	-0.815	0.477	0.091	0.145	0.305	0.637
Maximum degree of furcation: II	0.141	0.600	0.815	0.575	0.382	0.136
Maximum degree of furcation: III	2.297	1.056	0.035	0.534	0.666	0.427

#### Survival rate of splints

**Fig. 3:**
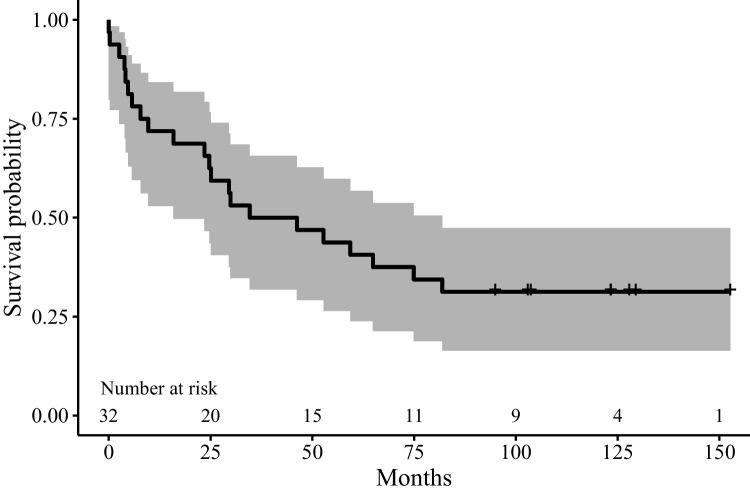
Kaplan–Meier estimator with 95% CI of time until fracture or debonding (splinting stability)

Ten years after T1 (up to 150 months after splint insertion), the survival rate of splints was 31%. Out of 32 splints, 22 had to be repaired during the observation period. Ten splints (in 7 patients) remained without the complication of a fracture or debonding. The Kaplan–Meier estimator of the survival rate of splints until fracture or debonding is shown in Fig. 3. Cox regression is used to test possible influencing factors on time to fracture/debonding. Due to the low number of test teeth with mobility degree I, the teeth with mobility degrees I and II are merged into one group. The same method is applied for Eichner classes B and C. Cox regression was not able to show an effect of the maximum mobility degree prior to splinting and the number of supporting zones on time to fracture/debonding (p = 0.212; p = 0.703). Since only two splints did not have antagonistic contact, this variable could not be used in the regression.

## Discussion

This study retrospectively investigates the changes in periodontal parameters of splinted compared to non-splinted posterior teeth and the survival rate of composite splints in the posterior during 10 years of SPT.

During the observation period of ten years of SPT, ten test teeth were lost, but only two control teeth. This can be attributed to the poorer initial periodontal condition of the splinted teeth, which had higher initial PPD, CAL and rABL at the beginning of the observation period. Although the periodontal baseline parameters at T1 were worse in the splinted teeth than in the non-splinted teeth, both groups showed similar low progression of mean PPD and mean CAL over the observation period of ten years SPT. A closer look at the individual PPD measurement sites at tooth level also shows that although the severity of periodontal disease in the test group is more pronounced, the change over the ten-year observation period is only slight. The number of teeth with deep pockets decreased by four in the test group, whereas the number of teeth with deep pockets remained unchanged in the control group.

The number of sites with BOP was also higher in the splinted compared to the non-splinted controls at T1, but these values also seem to be kept stable by regular SPT.

While almost all test teeth (96.6%) had pathological mobility prior to splint insertion (because of the inclusion criteria), this was not the case for the control teeth. Only 25.9% of the control teeth were diagnosed with pathological tooth mobility at the beginning of the observation period, and most of them had only a horizontal mobility up to 1 mm (mobility degree I). This difference could have influenced the results, especially since studies indicate that the initial degree of tooth mobility may have an influence on the periodontal therapy outcome [17] and since short-term results of a prospective study indicate a tendency for better periodontal therapy outcomes in splinted compared to non-splinted mobile mandibular anterior teeth [4, 18]. It would have been desirable to have control teeth with the same distribution of mobility degrees, but this could not be implemented in this retrospective observation due to the applied therapy concept (splint insertion in case of pathological tooth mobility). However, the test and control teeth were comparable with respect to the proportion and distribution of furcation involvement and the presence of direct antagonistic contacts (tooth-related).

With 2.92 ± 1.01 mm after 10 years of SPT (T2), the mean PPD of the splinted teeth is in a similar range as in another study that observed splinted anterior and posterior teeth (n = 227 teeth) over a similar period [5]. Thus, the PPD in Gratz et al. [5] was 3.2 ± 1.0 mm after a mean follow-up of 11 ± 7.2 years of SPT. The small change in periodontal parameters PPD and CAL observed at the splinted posterior teeth over a 10-year period of SPT in the present study, we also observed in a previous retrospective evaluation of these parameters over the same period at splinted mandibular anterior teeth [8]. In this study, the mean PPD on mandibular anterior teeth was significantly lower at 2.04 ± 0.47 mm, while the mean CAL was 5.0 ± 1.1 mm and thereby in a similar range compared to the splinted posterior teeth of the present study (5.52 ± 1.69 mm). The differences in PPD are probably since the control of the periodontal situation is easier on single-rooted teeth and in the anterior region than on multi-rooted teeth in the posterior [19]. For the change in mean PPD and CAL over ten years of SPT (ΔPPD and ΔCAL), an association was found with the respective baseline values at T1. The higher the PPD or CAL at T1, the greater the progression of the parameter over the subsequent ten years of SPT had been. Furthermore, the change in CAL (ΔCAL) was associated with complete furcation involvement (degree III). Teeth with furcation degree III showed the highest CAL increase over time. This result is consistent with other studies that showed that grade III furcation involvement leads to a significant worsening of prognosis [20, 21].

No influence was found for group affiliation (splinted vs. non-splinted teeth), as well as for the degree of mobility, the presence of systemic diseases at T1, smoking, and the presence of an antagonistic contact. The fact that smoking was not a factor influencing the progression of periodontal disease is worth mentioning, as smoking is one of the major risk factors for rapid progression of periodontitis. However, it can be argued that regular SPT based on individual periodontal risk could establish stable periodontal conditions even in smokers, or that the number of teeth included was simply too small to statistically demonstrate existing differences.

In the present study, 22 of the 32 included splints required at least one repair during the observation period, corresponding to a survival rate of the original splints of only 31% after ten years of SPT. Our results further indicate that the majority of first splint fractures occur within the first three years after splint insertion, with a flattening of the survival curve thereafter.

In a retrospective evaluation of splinted mandibular anterior teeth, also conducted in our department, the splint survival rate was 67% after 10 years of SPT. Kumbuloglu et al. [6] also found a remarkably high survival rate for splints in their prospective observation of 19 periodontitis patients that had splinting therapy from mandibular canine to canine. After 4.5 years the survival rate of splints was 94.8%. Thus, there appear to be significant differences between splinting stability in the mandibular anterior region compared to the posterior. This conclusion was also reached by Graetz et al. [5]. They investigated the long-term survival and maintenance efforts of splinted teeth in 57 periodontitis patients under SPT who had a total of 227 splinted teeth. The mean observation period of their study was 11.0 ± 7.2 years. 75% of all splints required at least one repair, which would correspond to a 25% survival rate of the original splints after eleven years of SPT. In the regression analysis, Graetz et al. [5] found that splints in the posterior region were more likely to fracture than splints in the mandibular anterior region. There was no association between the number of splint repairs required annually and the number of supporting zones, which is consistent with our results regarding the testing of possible factors influencing fracture probability, in which the number of supporting zones (according to Eichner) does not appear to be an influencing factor. In contrast to our study, Graetz et al. [5] used all non-splinted teeth of the dentition as control teeth and concluded that splinted teeth do not have a higher risk of loss than non-splinted teeth. It must be mentioned, however, that in Graetz et al. [5] 45.8% of the splinted teeth did not show any pathological mobility, whereas in the present study it is only 3.4%, but also a smaller number of included teeth.

Posterior teeth are exposed to higher chewing and shearing forces than anterior teeth and it can be assumed that this results in different loads on the splints in the different areas. This could influence the survival rate or the fracture probability, which would be a possible explanation for the different survival rates of splints in the posterior compared to the mandibular anterior region found in various studies. For this reason, the authors of the present study included only posterior teeth and, to further homogenize the test tooth group, included only splints that were placed due to mobility and excluded teeth that were treated resectively or regeneratively. Unfortunately, this resulted in a low number of study participants and included teeth, which is a limitation of the study.

The Cox regression on the survival probability of splinting does not identify the initial maximum degree of mobility of the teeth integrated into the splint or the number of supporting zones as influencing factors. Nevertheless, it should be mentioned that the Kaplan–Meier survival curve of the splints, distinguished by the maximum degree of mobility, for the splints containing a tooth with mobility degree III, has the lowest survival rate.

The study has several limitations, including the small number of patients and the retrospective study design. Furthermore, radiographs of suitable quality were not available for all test-control tooth pairs. The rABL was assessed from orthopantomograms or periapical radiographs using the same method, but this is still a potential limitation of the study. It would have been desirable to have the same type of standardized radiographs in all cases. Only patients compliant with SPT were included but it would be also interesting to compare the survival rate of splinted teeth and the splinting stability to non-compliant patients. Patients were defined as compliant if they attended at least one SPT session per year, regardless of their individual periodontitis risk. Future studies on this topic should also investigate the influence of the frequency of SPT sessions based on the individual periodontal risk profile or the degree of periodontitis on the stability of splinted teeth. Another limitation of the study is that, according to the inclusion criteria, in a small number of cases teeth with no pathological mobility or low mobility were also included as test teeth, for example if they were included in the splint because of the high mobility of the adjacent teeth. It should also be discussed that a case–control design of the study and using non-splinted teeth with similar periodontal damage as controls would also be adequate to investigate the change in periodontal parameters of splinted versus non-splinted teeth. An BL, the severity of periodontal disease is more pronounced in the group of test teeth than in the group of control teeth. However, the study clearly addresses the issue of periodontal stability, defined as the change in periodontal parameters over the observation period. Due to the split-mouth design, test and control teeth are matched for the systematic periodontal treatment applied, the professional and home oral hygiene measures and all possible patient-level factors (e.g. general diseases, smoking). This is an advantage compared to a case control study. Finally, it must be mentioned that all included patients were treated by periodontal specialist in a university setting and therefore results cannot be generalized.

## Conclusions

Despite the limitations of the study, it can be concluded that splinting does not provide any disadvantage in terms of the overall periodontal situation of periodontally damaged posterior teeth with increased mobility. However, splint fractures seem to occur very often in the posterior region. Although the repair or new insertion of a composite or composite fibre-reinforced splint is not time consuming and of low costs, the dentist should be aware of the high fracture probability of the splint and inform the patient accordingly that frequent repairs may be needed.

## Data Availability

No datasets were generated or analysed during the current study.
